# Effective dose of intravenous oxycodone depending on sex and age for attenuation of intubation-related hemodynamic responses

**DOI:** 10.3906/sag-2004-63

**Published:** 2021-02-26

**Authors:** Keum Young SO, Ki Tae JUNG, Bo Hyun JANG, Sang Hun KIM

**Affiliations:** 1 Department of Anesthesiology and Pain Medicine, Chosun University Hospital, Gwangju Republic of Korea; 2 Department of Anesthesiology and Pain Medicine, School of Medicine, Chosun University, Gwangju Republic of Korea; 3 Department of Medicine, Graduate School of Chosun University, Gwangju Republic of Korea

**Keywords:** Hemodynamics, humans, intravenous administration, intubation, laryngoscopy, oxycodone

## Abstract

**Background/aim:**

Preoperative intravenous oxycodone may help to prevent or attenuate intubation-related hemodynamic responses (IRHRs), but its pharmacokinetics differs according to age and sex. Therefore, we investigated the 95% effective dose (ED_95_) of intravenous oxycodone for attenuating all IRHRs, depending on the age and sex of the study population.

**Materials and methods:**

All patients were allocated to one of 6 groups: 1) 20–40 year old males, 2) 41–65yearold males, 3) 66–80 year old males, 4) 20–40 year old females, 5) 41–65yearold females, and 6) 66–80 year old females (groups YM, OM, EM, YF, OF, and EF, respectively). Using Dixon’s up-and-down method, the first patient in each group was slowly injected with intravenous oxycodone (0.1 mg kg^-1^) 20 min before intubation. The subsequent patient received the next oxycodone dose, which was decreased or increased by 0.01 mg kg^-1^, depending on the “success” or “failure” of attenuation of all IRHRs to within 20% of the baseline values at 1 min after intubation in the previous patient. After obtaining 8 crossover points, predictive ED_95_ was estimated with probit regression analysis.

**Results:**

ED_95_ varied greatly according to age and sex. ED_95_was 0.133 mg kg^-1^, 0.181 mg kg^-1^, 0.332 mg kg^-1^, 0.183 mg kg^-1^, 0.108 mg kg^-1^, and 0.147 mg kg^-1^in groups YM, OM, EM, YF, OF, and EF, respectively.

**Conclusion:**

ED_95_ is higher in males with increasing age but is ambiguous for females. ED_95_ is higher in males than in females over 40 years of age but is higher in females than in males under 41 years of age. However, after considering the age and sex of the study population, these results can be used as reference doses for further studies to verify the clinical effects of oxycodone for attenuating all IRHRs.

## 1. Introduction

Direct laryngoscopy and endotracheal intubation have a risk of undesirable hemodynamic changes, which may lead to potentially fatal events like cardiac arrhythmia, cardiac failure, or cerebral hemorrhage, especially in patients with cardiovascular or cerebral disease. Therefore, various drug interventions with local anesthetics, opioids, and other cardiovascular medicines have been used to attenuate intubation-related hemodynamic responses (IRHRs) [1].

Opioids are used for preoperative pain control in approximately 23.1% of patients, most commonly for those undergoing orthopedic and neurosurgical procedures [2]. Hydrocodone (59.4%), tramadol (21.2%), and oxycodone (18.3%) are the most commonly used opioids [2]. Among them, oxycodone has been reported to be beneficial in reducing intraoperative stress reactions, postoperative pain scores, and postoperative rescue analgesic consumption when it is administrated in the preoperative period [3–5]. 

Based on the reduction of intraoperative stress reactions by oxycodone, we hypothesized that preoperative intravenous oxycodone may help to prevent or attenuate IRHRs. Several studies were conducted to determine the effective dosage of oxycodone [6–8]. However, despite the pharmacokinetics of oxycodone varying according to age and sex [9–11], the oxycodone doses suggested by these studies have been determined regardless of age and/or sex [6–8].

We investigated the 95% effective dose (ED_95_) of intravenous oxycodone, which could attenuate all IRHRs to within 20% changes of the baseline values, depending on the age and sex of our study population. 

## 2. Materials and methods

We enrolled patients who were between 20–80 years old, who had an American Society of Anesthesiologists (ASA) physical status of I or II, and who were scheduled to undergo elective surgery under general anesthesia. We excluded patients with chronic opioid medication, past history of opioid-related complications, recent medication with a monoamine oxidase inhibitor, renal or hepatic function abnormalities, cardiopulmonary disease, neurovascular disease, mental disorders, and all patients with an ASA status ≥III. We also excluded those patients for whom successful intubation took longer than 15 s and all other participants who were deemed vulnerable by the IRB. 

All patients were allocated to 1 of 6 groups according to sex and age: 1) 20–40 year old males (group YM), 2), 41–65 year old males (group OM), 3) 66–80 year old males (group EM), 4) 20–40 year old females (group YF), 5) 41–65 year old females (group OF), and 6) 66–80 year old females (group EF). 

After premedication with intramuscular midazolam (0.05 mg kg^-1^), the patients were transported to an operating room. Prior to the induction of anesthesia, standard patient monitoring devices to obtain electrocardiograms, noninvasive blood pressure, end-tidal partial pressure of carbon dioxide, and peripheral pulse oximetry were applied. We defined the baseline hemodynamic values as the first values obtained after the patient was taken into the operating room. The radial artery was cannulated to obtain continuous blood pressure monitoring. Patients were premedicated with 0.3 mg ramosetron under preoxygenation with O2 6 L min–1 via a face maskto prevent postoperative nausea and vomiting.

**Figure 1:**
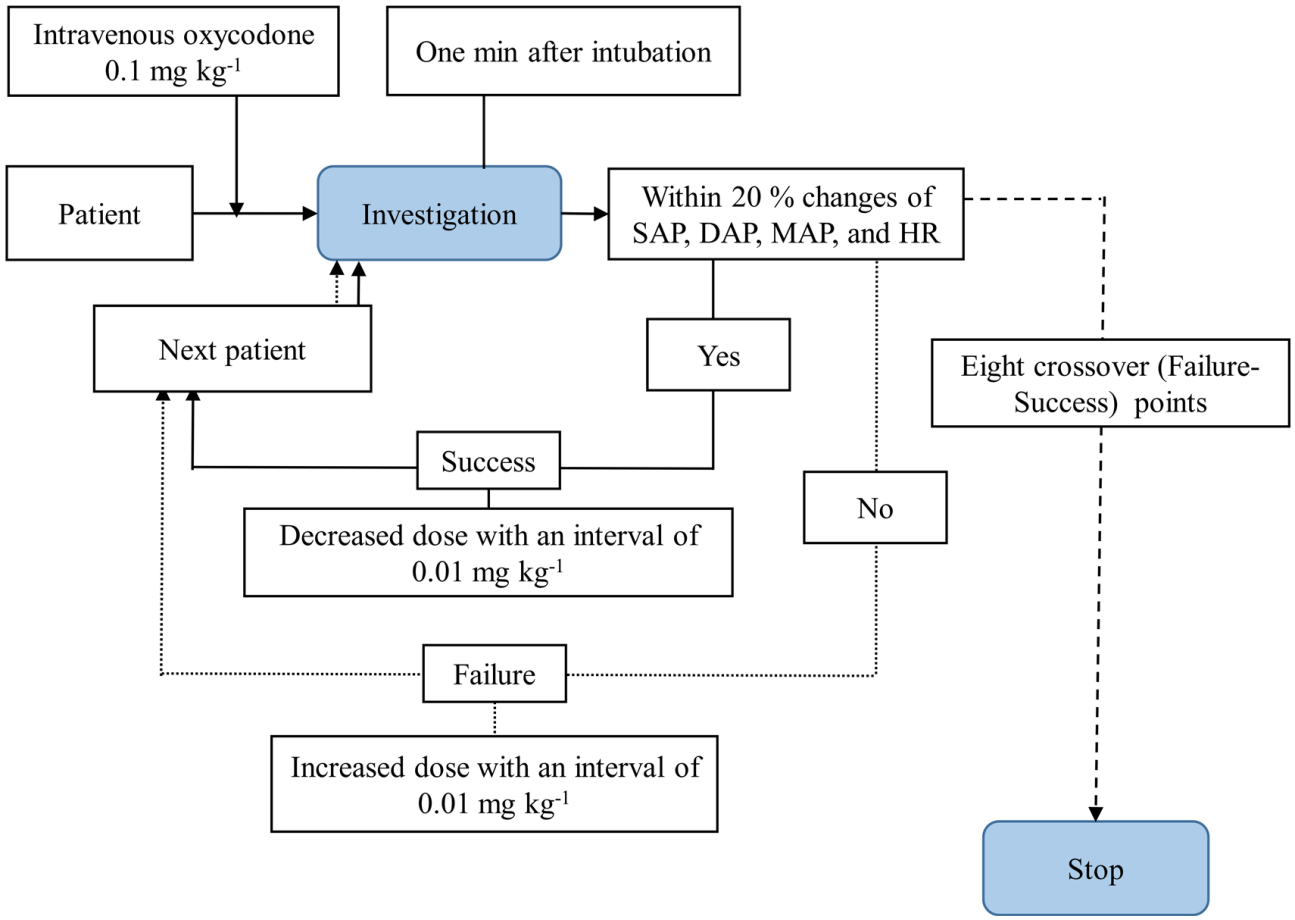
Diagrammatic representation of Dixon’s up-and-down method. SAP: systolic arterial pressure; DAP: diastolic arterial pressure; MAP: mean arterial pressures; HR: heart rate.

We used Dixon’s up-and-down method (Figure 1). The first patient in each group was slowly injected with intravenous oxycodone 0.1 mg kg^-1^ (over 2 min) in the presence of an experienced anesthesiologist who closely monitored each patient and ensured their safety and subsequently intubated them 20 min later. After initiation of mask ventilation with a 50% oxygen–air mixture, intravenous propofol (2 mg kg^-1^) was administered, followed by the monitoring of anesthetic depth monitoring (BIS or entropy) and neuromuscular monitoring. Endotracheal intubation was performed 2 min after the injection of rocuronium (0.9 mg kg^-1^) [12]. “Success” or “failure” of the effect of oxycodone was determined 1 min after intubation, based on whether the IRHRs significantly differed from their baseline values. We defined “success” as a ≤20% change in all of the parameters monitored, namely the systolic, diastolic, mean arterial pressures (SAP, DAP, and MAP, respectively), and heart rate (HR); “failure” was defined as a >20% change in any of these parameters [12]. The subsequent patient received the next oxycodone dose, which was decreased or increased by 0.01 mg kg^-1^, depending on the “success” or “failure” of the previous patient, respectively [12,13]. We repeated Dixon’s up-and-down method until we obtained 8 crossover points, which were manifested as crossover from “failure” to “success” [12].

Patients and investigators were blinded to the study medication, which consisted of the oxycodone dose for the next patient according to the response of the previous patient. A noninvestigating nurse loaded the doses into indistinguishable numbered syringes with the same volume (5 mL by mixing with normal saline). The syringes were delivered to the attending anesthesiologist, who was allocated with a random table generated by a computer, 25 min before intubation. After endotracheal intubation, the response was recorded on the injected study medication andwhether the dose for the next patient would be decreased or increased was determined. Then, the case report form was delivered to the noninvestigating nurse.

The age, sex, height, weight, body mass index (BMI), ASA physical status, SAP, DAP, MAP, and HR was recorded for each patient. 

### 2.1. Statistical analysis

The primary endpoint was the predictive ED_95_ of intravenous oxycodone for attenuating all IRHRs 1 min after intubation. SPSS (Windows ver. 26.0, IBM Corp., Armonk, NY, USA) was used for statistical analysis. All measured values of demographic data are presented as mean (95% confidence intervals [95% CI]) or as the number of patients. 

The chi-square test was used for the analysis of sex and ASA physical status. The one-way ANOVA, followed by the Scheffe posthoc test, were used for the analysis of age, height, weight, and BMI. 

The calculated ED_50_ of intravenous oxycodone was the mean value of the midpoint doses of all independent pairs of patients who manifested as a crossover from “failure” to “success” after 8 crossover points in each group by Dixon’s upanddown method [12]. The calculated ED_50_ of intravenous oxycodone are presented as means (95% CI). The Kruskal–Wallis test, followed by automatic pairwise comparisons after Bonferroni correction, was used for analysis of the calculated ED_50_ of oxycodone. P values <0.05 were considered to be statistically significant.

The predictive ED_50_ and ED_95_ of intravenous oxycodone were estimated with a probit regression model. The predictive ED_50_ and ED_95_ of intravenous oxycodone are presented as predictive values (95% CI). 

## 3. Results

**Figure 2:**
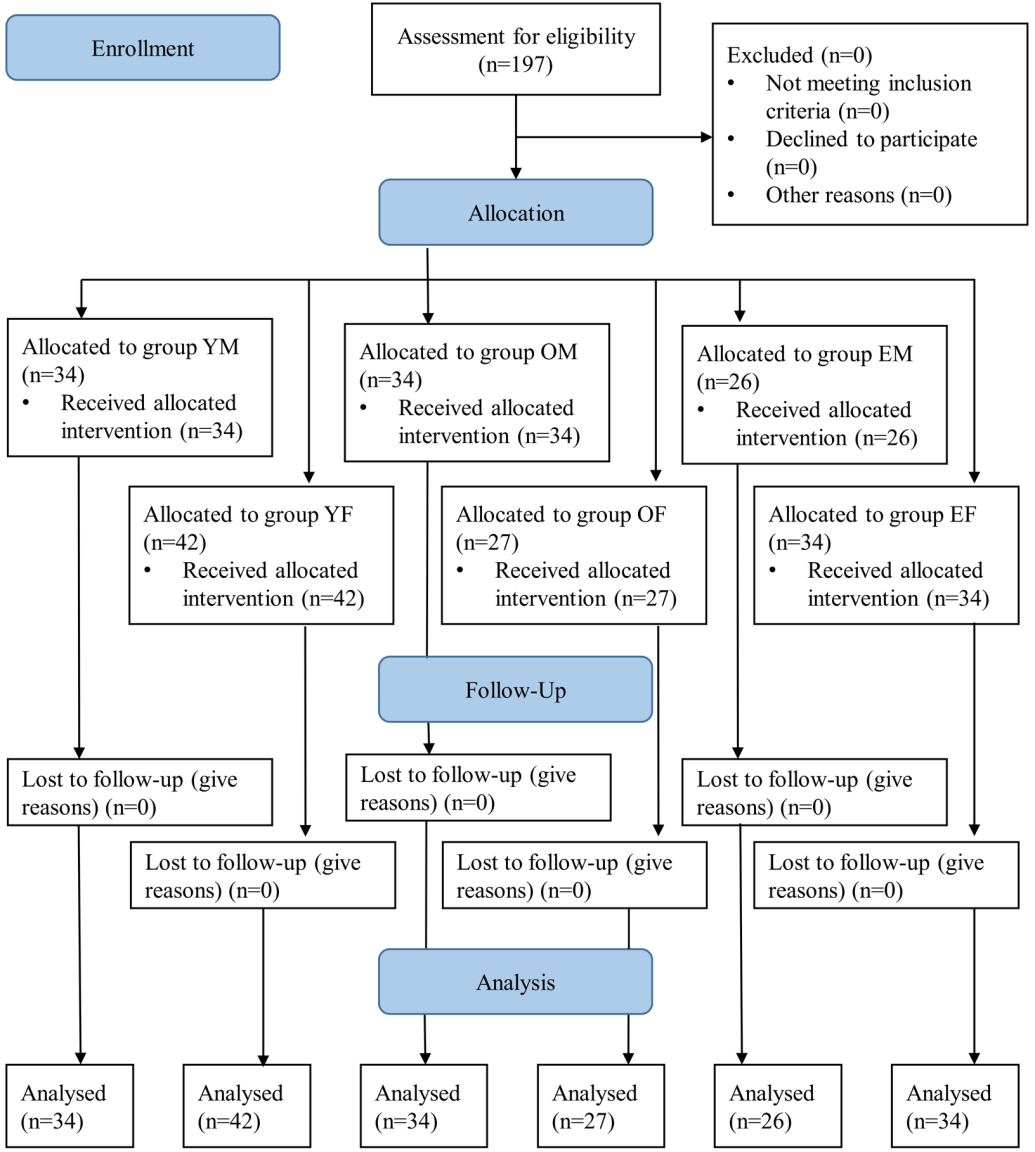
CONSORT (Consolidated Standards of Reporting Trials) diagram. Group YM: males between 20 and 40 years old; group OM: males between 41 and 65 years old; group EM: males between 66 and 80 years old; group YF: females between 20 and 40 years old; group OF: females between 41 and 65 years old; group EF: females between 66 and 80 years old.

A total of 197 patients (34 patients in group YM, 34 in group OM, 26 in group EM, 42 in group YF, 27 in group OF, and 34 in group EF) were finally enrolled after Dixon’s upanddown method (Figure 2).

**Figure 3:**
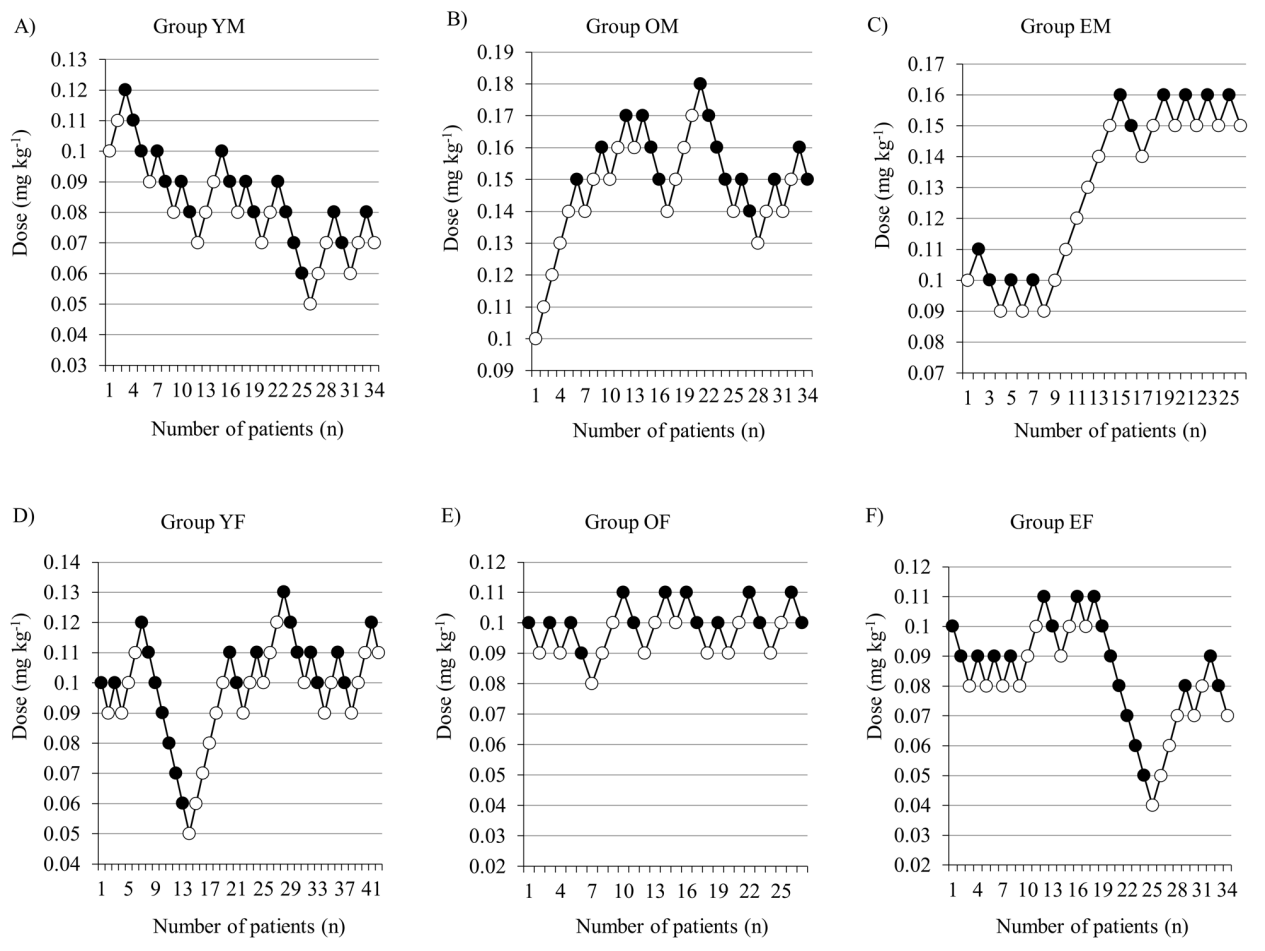
Consecutive dose of intravenous oxycodone during Dixon’s up-and-down. Figures show “success” or “failure” of the effect of oxycodone 1 min after intubation in group YM (a); group OM (b); group EM (c); group YF (d); group OF (e); and group EF (f). White circles: “failure”; black circles: “success”.

**Figure 4:**
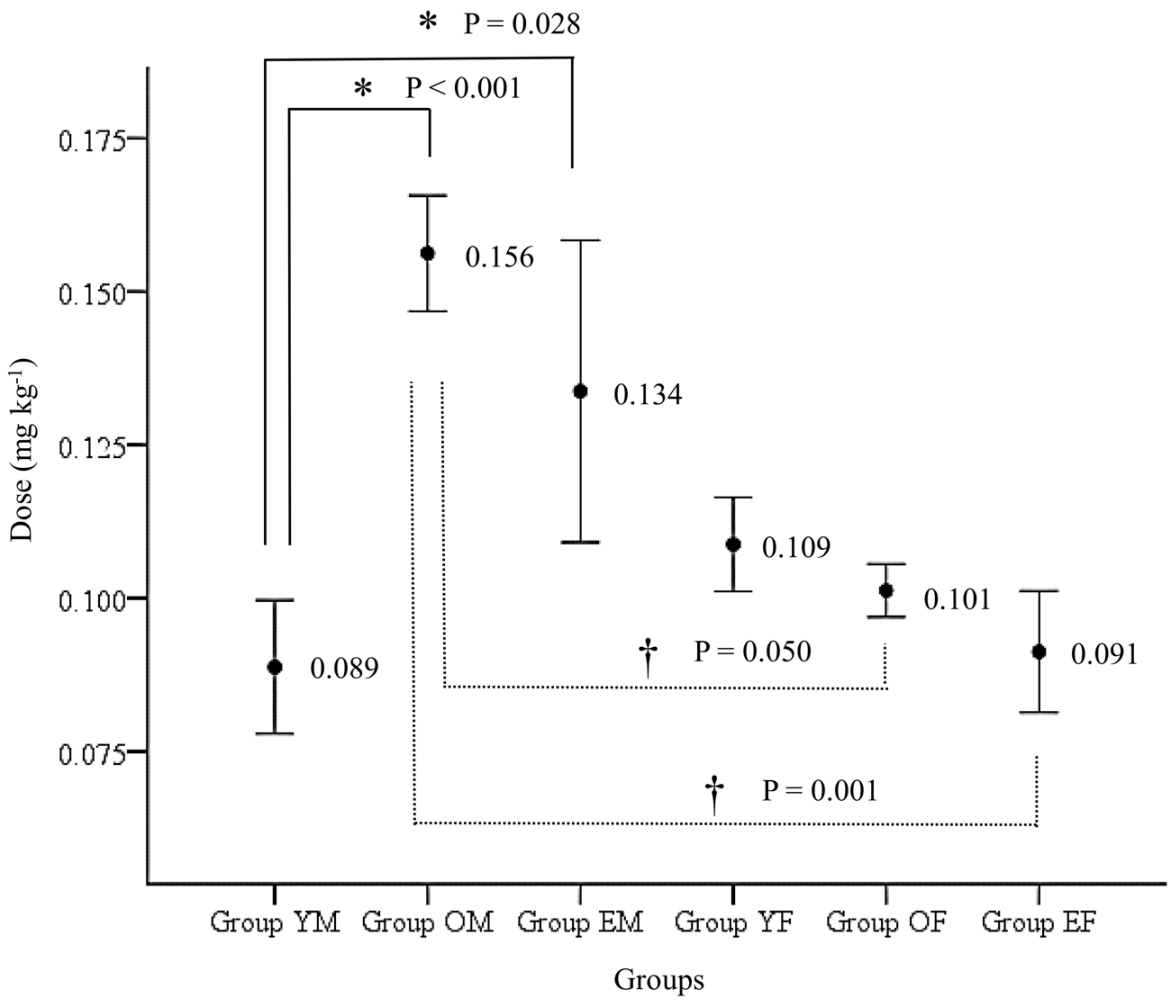
ED_50_ calculated from Dixon’s up-and-down method depending on sex and age. The calculated ED_50_of oxycodone was 0.089 (95% CI, 0.078–0.100) mg kg^-1^, 0.156 (95% CI, 0.147–0.166) mg kg^-1^, 0.134 (95% CI, 0.109–0.158) mg kg^-1^, 0.109 (95% CI, 0.101– 0.116) mg kg^-1^, 0.101 (95% CI, 0.097–0.106) mg kg^-1^, and 0.091 (95% CI, 0.081–0.101) mg kg^-1^ in groups YM, OM, EM, YF, OF, and EF, respectively. CI: confidence interval;ED_50_: effective dose of intravenous oxycodone that could attenuate all intubation-related hemodynamic response changes to less than 20% over baseline values in 50% of patients. *: P < 0.05, compared with group YM; †:P <0.05, compared with group OM.

There were significant differences in age, sex, height, weight, and ASA physical status but not in BMI among the groups (Table), but this was expected as the patients had been grouped in terms of age and sex for the study. The ED_50_ of oxycodone, which was calculated using Dixon’s upanddown method, was 0.089 (95% CI, 0.078–0.100) mg kg^-1^, 0.156 (95% CI, 0.147–0.166) mg kg^-1^, 0.134 (95% CI, 0.109–0.158) mg kg^-1^, 0.109 (95% CI, 0.101–0.116) mg kg^-1^, 0.101 (95% CI, 0.097–0.106) mg kg^-1^, and 0.091 (95% CI, 0.081–0.101) mg kg^-1^ in groups YM, OM, EM, YF, OF, and EF, respectively (Figures 3a–3f and 4). The calculated ED_50_ in group YM was significantly lower than that in groups OM and EM (P < 0.001 and P = 0.028, respectively). The calculated ED_50_ in groups YF, OF, and EF did not show any significant differences between the groups. The calculated ED_50_ in group OM was significantly higher than that in groups OF and EF (P = 0.050 and P = 0.001, respectively). There were no significant differences between groups YM vs. YF, and groups EM vs. EF.

**Table:** Demographic and intraoperative data.

	Group YM(n = 34)	Group OM(n = 34)	Group EM(n = 26)	Group YF(n = 42)	Group OF(n = 27)	Group EF(n = 34)	P value
Age (years)	29.4(27.2–31.6)	52.8(50.1–55.4)	73.5(71.8–75.2)	27.7(25.7–29.7)	54.6(51.6–57.7)	73.6(71.9–75.3)	< 0.001
Sex (M/F)	34/0	34/0	26/0	0/42	0/27	0/34	< 0.001
Height (cm)	173.4(171.7–175.0)	168.6(166.6–170.6)	167.2(164.6–169.8)	161.7(160.1–163.4)	156.3(154.3–158.3)	152.3(150.3–154.3)	< 0.001
Weight (kg)	74.4(70.2–78.5)	68.2(65.4–71.1)	64.9(61.3–68.4)	63.2(58.7–67.7)	59.3(55.6–63.1)	55.5(52.0–58.9)	< 0.001
BMI (kg (m2) -1)	24.67(23.48–25.86)	24.02(23.04–25.00)	23.19(22.07–24.31)	24.12(22.51–25.73)	24.22(22.99–25.44)	23.87(22.57–25.17)	0.777
ASA_PS (I/II)	32/2	23/11	4/22	34/8	13/14	5/29	< 0.001

The values are expressed as mean (95% confidential intervals) or number of patients. ASA_PS: American Society of Anesthesiologists physical status; BMI: body mass index. P < 0.05 was considered to indicate statistical significance.

**Figure 5:**
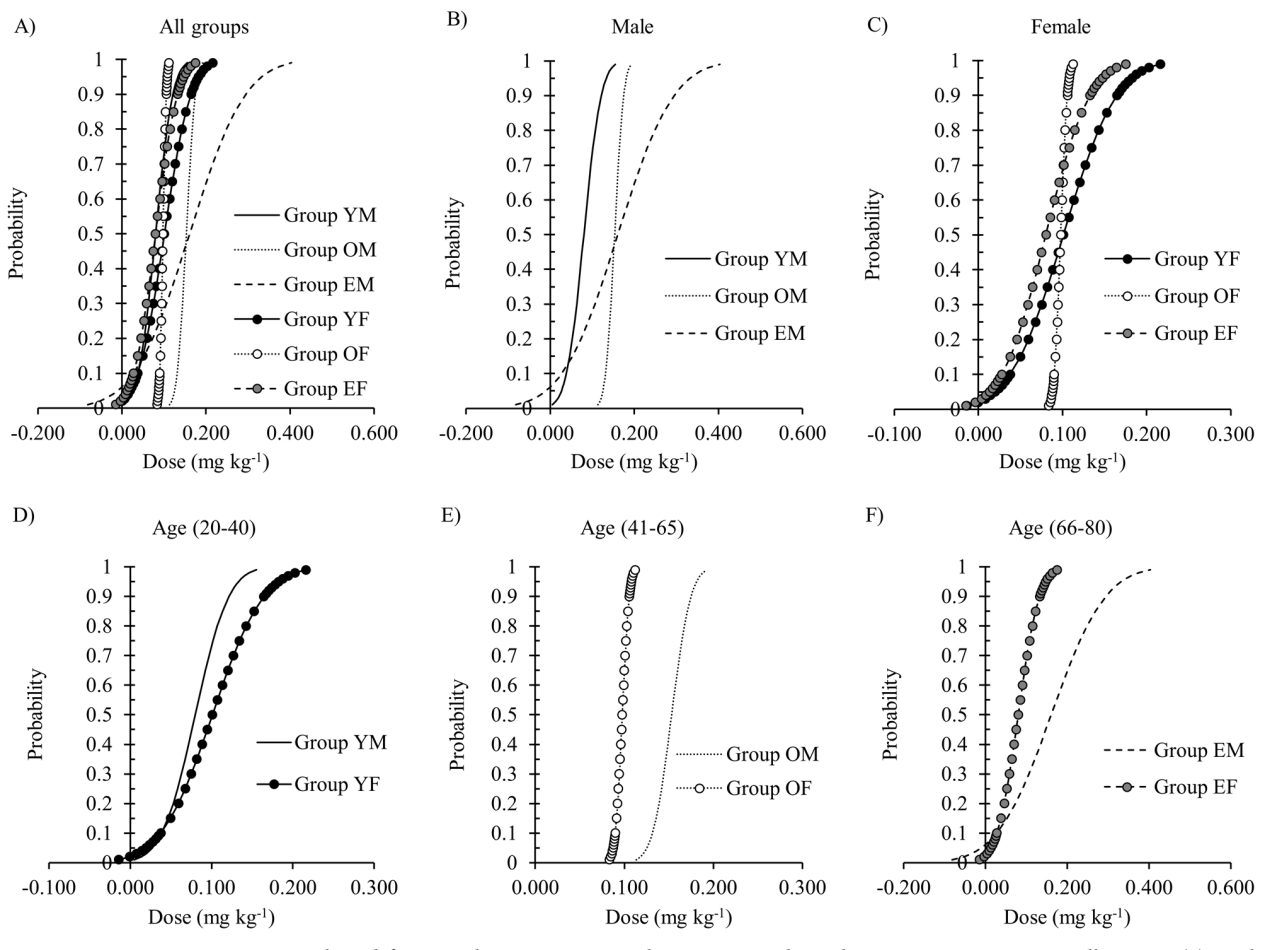
Dose response curves plotted from probit regression analysis. Figures show dose response curves in: all groups (a); male groups (b); female groups (c); age groups 20 to 40 years (d); age groups 41 to 65 years (e); age groups 66 to 80 years (f).

**Figure 6:**
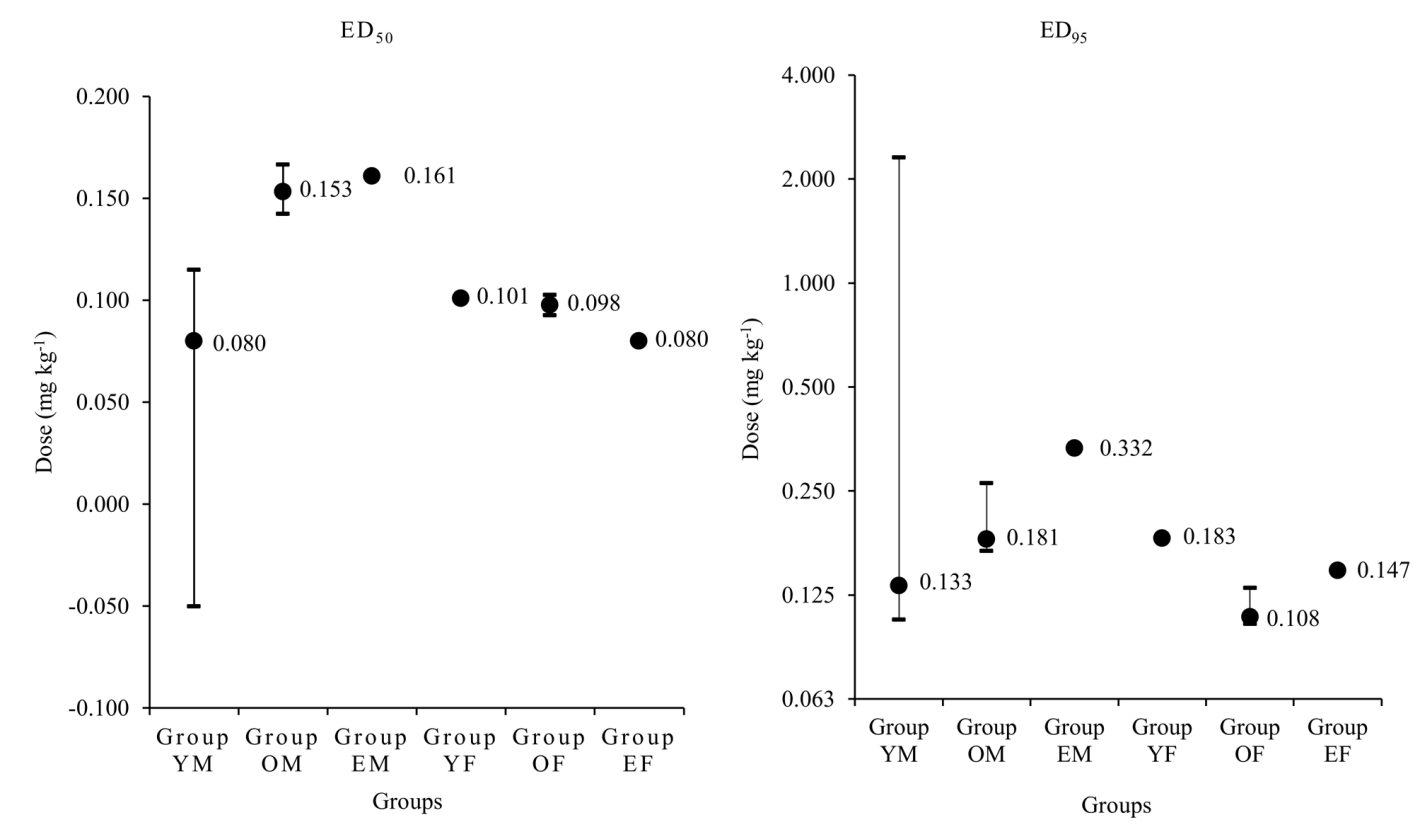
Predictive oxycodone ED_50_ and ED_95_ from the probit regression model.The predictive oxycodone ED_50_ was 0.080 [95% CI, (–0.050) –0.115] mg kg^-1^, 0.153 (95% CI, 0.142–0.166) mg kg^-1^, 0.161 (95% CI, incomputable) mg kg^-1^, 0.101 (95% CI, incomputable) mg kg^-1^, 0.098 (95% CI, 0.093–0.103) mg kg^-1^, and 0.080 (95% CI, incomputable) mg kg^-1^ in groups YM, OM, EM, YF, OF, and EF, respectively. The predictive oxycodone ED_95_ was 0.133 (95% CI, 0.106–2.305) mg kg^-1^, 0.181 (95% CI, 0.167–0.263) mg kg^-1^, 0.332 (95% CI, incomputable) mg kg^-1^, 0.183 (95% CI, incomputable) mg kg^-1^, 0.108 (95% CI, 0.103–0.129) mg kg^-1^, and 0.147 (95% CI, incomputable) mg kg^-1^ in groups YM, OM, EM, YF, OF, and EF, respectively. CI: confidence interval; ED_50_, ED_95_: effective dose of intravenous oxycodone that could attenuate all intubation-related hemodynamic response changes to less than 20% over baseline values in 50% and 95% of patients, respectively.

The predictive oxycodone ED_50_ from the probit regression model was estimated as 0.080 [95% CI, (–0.050)–0.115] mg kg^-1^, 0.153 (95% CI, 0.142–0.166) mg kg^-1^, 0.161 (95% CI, incomputable) mg kg^-1^, 0.101 (95% CI, incomputable) mg kg^-1^, 0.098 (95% CI, 0.093–0.103) mg kg^-1^, and 0.080 (95% CI, incomputable) mg kg^-1^ in groups YM, OM, EM, YF, OF, and EF, respectively (Figures 5a–5f and 6).

The predictive oxycodone ED_95_ from the probit regression model was estimated as 0.133 (95% CI, 0.106–2.305) mg kg^-1^, 0.181 (95% CI, 0.167–0.263) mg kg^-1^, 0.332 (95% CI, incomputable) mg kg^-1^, 0.183 (95% CI, incomputable) mg kg^-1^, 0.108 (95% CI, 0.103–0.129) mg kg^-1^, and 0.147 (95% CI, incomputable) mg kg^-1^ in groups YM, OM, EM, YF, OF, and EF, respectively (Figures 5a–5f and 6).

## 4. Discussion

This study aimed to evaluate the ED_50_ and ED_95_of intravenous oxycodone for the attenuation of all IRHRs to within 20% changes of the baseline values, depending on sex and age of the study population, rather than determine the clinical effects of these doses. The doses varied (ED_50_: 0.080–0.161 mg kg^-1^, ED_95_: 0.108–0.332 mg kg^-1^) according to age and sex. The ED_50_ and ED_95_ of oxycodone were found to be higher in male patients with increasing age but were ambiguous in female patients. 

Several studies have suggested that the effective dose of oxycodone to minimize IRHRs was above 0.1 mg kg^-1^[6–9,12], and intravenous oxycodone above 0.14 mg kg^-1^ was as effective as fentanyl 2 µg kg^-1^, with a lower complication rate of apnea and less postoperative pain [7,8,14]. Park et al. [6] found that predictive ED_50_ andED_95_ of intravenous oxycodone, obtained with probit analysis focused on blood pressure, were 0.020 mg kg^-1^ and 0.159 mg kg^-1^, respectively, for preventing IRHRs in healthy patients. Our previous study in male patients also showed that oxycodone 0.182 mg kg^-1^ was as effective as fentanyl 2 µg kg^-1^ despite the predictive ED_95_ of intravenous oxycodone being 0.091 mg kg^-1^ in attenuating all IRHR changes to less than 20% over the baseline 1 min after intubation [12]. However, these doses were not sufficient for attenuating all IRHRs, including heart rate changes. Intravenous oxycodone below 0.2 mg kg^-1^ was not effective in attenuating all IRHRs within a 15% increase from the baseline [6], and probit analysis with the data of our previous study also showed that the recalculated oxycodone ED_95_ was 0.269 mg kg^-1^ to prevent all IRHR changes in male patients [12]. Therefore, intravenous oxycodone above 0.2 mg kg^-1^ may be required to attenuate all IRHRs, including heart rate changes [9]. 

Oxycodone is known to demonstrate variable pharmacokinetics that is greatly dependent on age and sex. The blood concentration, sensitivity, metabolic capacity, and bioavailability of oxycodone are higher in females than in males [9–11,15]. With increasing age, oxycodone has greater bioavailability and lower total body clearance from plasma [10,11,15]. Therefore, it seems reasonable to titrate the dose of oxycodone on an individual basis, particularly in older adults aged above 70 years and in female patients. A previous clinical study also suggested that the ED_50_ and ED_95_ of intravenous oxycodone for blunting all IRHRs in adult patients (18–64 years old) were affected by sex and that male patients (0.324 mg kg^-1^ and 0.454 mg kg^-1^) required 28% more intravenous oxycodone than female patients (0.254 mg kg^-1^ and 0.357 mg kg^-1^) [9]. In this study, the predictive ED_95_ of intravenous oxycodone increased in male patients with increasing age but not in female patients. The predictive ED_95_ was higher in older and elderly male patients than in female patients [9]. However, younger patients (between 20–40 years old) had a higher predictive ED_95_ in females than in male patients. 

It is difficult to compare our results with those of previous reports because of study complexities with different cut-off values (15% vs. 20% change from baseline), different starting doses for Dixon’s up-and-down method, and variable study designs (Dixon’s up-and-down method vs. a randomized controlled study). Firstly, the larger cut-off value (<20%) used in this study may result in a smaller effective dose of oxycodone compared with that of studies that used a 15% change as the cut-off value, which is a more strict criterion. 

Second, Dixon’s up-and-down method demands that the starting dose should be the minimum dose expected to result in a positive response [16]. If the choice of the starting dose is inappropriate, a string of repeated identical outcomes may occur; eventually, the ED_50_ may be much lower or higher than the starting dose. However, this optimal minimum dose was not known. Therefore, as a starting dose, we used 0.1 mg kg^-1^ of intravenous oxycodone, considering the predictive ED_95_ (0.091 mg kg^-1^) obtained from Goh’s study [12]. Kang et al. opted for 0.2 mg kg^-1^ in female patients, which was an effective dose used for the randomized controlled trial studies [7,8] and a larger dose (0.30 mg kg^-1^) in male patients [9]. Thus, even though the same cut-off value might be used, different starting doses can influence the effective dose obtained with probit regression from the results of Dixon’s up-and-down method. 

Third, the predictive ED_50_ and ED_95_ calculated by Dixon’s up-and-down method and the randomized controlled trial method may be different. Our previous study in male patients showed that the predictive ED_95_ of oxycodone calculated with data from Dixon’s up-and-down method was 0.091 mg kg^-1^, but its actual preventing effect was 62.2% [12]. From the randomized controlled study with this predictive ED_95_, the recalculated predictive ED_95_ was 0.269 mg kg^-1^for attenuating all IRHRs, 0.152 and 0.188 mg kg^-1^ for attenuating changes of SAP and MAP, respectively, and0.217 mg kg^-1^ for attenuating changes in HR [12]. It was within the 95% CI of ED_95_ for attenuating the changes of SAP and MAP in Park’s study, using less than 15% change as a cut-off level for individual IRHRs [6].

There are several limitations of this study, as well. First, we did not exclude hypertensive patients in the operating room if the patients were of ASA physical status I or II and were not on any cardiac or antihypertensive medications. Hypertensive patients are associated with a marked increase in plasma noradrenaline concentration and a moderate increase in adrenaline concentration after intubation, while normotensive patients experience only a moderate increase in plasma noradrenaline concentration without an accompanying change in the plasma adrenaline concentration [17]. Therefore, hypertensive patients show a transient increase in sympathetic activity as a result of increased sensitivity of peripheral vessels and due to the increased secretion of catecholamines in response to intubation [18,19]. Consequently, IRHRs are exaggerated and variable in hypertensive patients when compared to normotensive patients [18,19]. Second, data interpretation should be performed from a perspective of the 95% confidence interval rather than the mean or representative values, even though the representative values of ED_50_ and ED_95_ seem to be significantly different or undocumented. If the 95% CIs overlap considerably or come very close together, this indicates that there is little or no probability of obtaining a significant differenceat the P = 0.05 level. In Kang’s study [9], most of the values had 95% CIs that were either overlapping considerably or quite close together. Furthermore, this study showed some results with incomputable 95% CIs. Thus, it was not possible to interpret the significance of these results, if any. 

Opioid-induced complications such as hypotension, bradycardia, and respiratory depression are also important perioperative concerns at high intravenous doses of oxycodone. However, doses of up to 0.2 mg kg^-1^of oxycodone have shown comparable complications with fentanyl 2 µg kg^-1^[7,8].In our study, we did not observe any oxycodone-related adverseeffects with its slow administration over 2 min, even at the maximum dose of 0.16 mg kg^-1^. Additionally, our patients were on supplemental oxygen (50% oxygen–air mixture) through face masks until they were intubated.

In conclusion, the ED_50_ and ED_95_ of intravenous oxycodone for the attenuation of all IRHRs to within 20% changes of the baseline values varied (ED_50_: 0.080–0.161 mg kg^-1^, ED_95_: 0.108–0.332 mg kg^-1^) according to the age and sex of the study population. The ED_50_ and ED_95_ of oxycodone were higher in male patients with increasing age but were ambiguous in female patients. In addition, ED_50_ and ED_95_ of oxycodone were higher in males compared with females under 40 years of age but higher in females compared with males over 41 years of age. However, these dose variations according to the age and sex of the study population were not statistically significant, and the clinical impact of these results on the attenuation of all IRHRs, if any, has yet to be ascertained. Further studies are needed to verify the clinical effects and complications of multiple ED_95_ of oxycodone according to age and sex.

## Informed consent

This prospective, randomized, controlled, and double-blinded study was approved by the Institutional Review Board of Chosun University Hospital (Chosun 2017-03-007) and was registered with the Clinical Research Information Service (CRIS, Ref: KCT0002491) on August 2, 2017. 

Written informed consent was obtained from all participants or a legal surrogate. This study was conducted according to the Declaration of Helsinki 1964 and all subsequent revisions.
